# Ready References to Amendments, Revocations, Modifications, Explanations, Etc.

**Published:** 1919-01

**Authors:** 


### READY REFERENCES TO AMENDMENTS, REVOCATIONS, MODIFICATIONS, EXPLANATIONS, ETC.

**As Amended to Include November 8, 1918.**

From G. O. 1 to 180 dated Oct. 15, 1918.

Bul. 1 to 78 dated Oct. 15, 1918.

Prepared by

LEGAL REFERENCE SECTION,

Finance and Accounting Division.

C. S. O. A. E. F.

### INDEX
### to
### GENERAL ORDERS AND BULLETINS
### G. H. Q. 1918

| PURPORTS | G. O. | Bul. | Sec. | Par. |
|---|---|---|---|---|
| **Absence —** | | | | |
| Other than on duty or leave. . . . . . . | 6 | | I | 4 |
| Without leave over 24 hours. . . . . . . | 25<br>56 | | I | 2<br>4 |
| **Absentees —** | | | | |
| Reporting of, to nearest Provost Marshal. | 25 | | I | 2 |
| Apprehension and return of . . . . . . . | 113 | | IV | |
| **Accidents, Motor Vehicles —** | | | | |
| Report and investigation of . . . . . . . | 11 | | III | |
| Action to be taken after. . . . . . . . . | 43 | | IV | 3 (j) |
| **Accountable Officers —** | | | | |
| Before Discharge of the A. E. F. must comply with the regulations of this order. . . . . . . . . . . . . . . . . | 127 | | I | |
| **Accountability —** | | | | |
| For animals. . . . . . . . . . . . . . . . | 122 | | IX | 7. |
| For Ordnance and arms equipment. . . . | 150 | | III | |
| Property, for Officers. . . . . . . . . . | 167 | | IV | |
| **Addresses —** | | | | |
| Manner of writing. . . . . . . . . . . . | 61 | | | 5 (d) |
| Of enlisted men while on leave . . . . . | 6 | | | |
| Of allottee or beneficiary, change in. . . | | 77 | | |
| Rules governing. . . . . . . . . . . . . | 146 | | IV | |
| **Addressing Mail —** | | | | |
| Or telegraphic communications to units and individuals in the A. E. F. . . . | 155 | | III | |
| **Adjutant General, A. E. F. —** | | | | |
| Charged with. . . . . . . . . . . . . | 100 | | | |
| **Advance Supply Depot —** | | | | |
| To receive excess property. . . . . . . . | 10 | | II | |
| **Age Limit —** | | | | |
| Minimum for Army Candidate School. . . | 121 | | III | 1 |
| **Agencies of Supply and Evacuation —** | | | | |
| In the Advance Section, S. O. S. | 44 | | | 15-22 |
| **Aix-les-Bains —** | | | | |
| Leave center for soldiers. . . . . . . . | 38 | | | 1 and 3 |
| **Alcohol —** | | | | |
| Consolidated, when used . . . . . . . . | 117 | | I | 5 |
| **Allied Armies —** | | | | |
| Sales of subsistence to Officers and Enlisted Men on duty with A. E. F. . . | 159 | | II | |

| PURPORTS | G. O. | Bul. | Sec. | Par. |
|---|---|---|---|---|
| **Aliens —** | | | | |
| In military service of the U. S. A., Law and rules for Bureau of Naturalization. | | 68 | | |
| May make request to change name upon application for naturalization. . . . . . | | 77 | | |
| Naturalization of —, form to be used. . . | 151 | | | |
| To show Army serial number and petition number upon applying for naturalization. | 171 | | I | |
| **Allotments —** | | | | |
| Abstracts of. . . . . . . . . . . . . . . . | | 31 | | |
| Change of address of allottee to be forwarded through certain channels. . . . | | 77 | | |
| Enlisted men's pay, disposition of. . . . . | 16 | | II | |
| Enlisted men who have made allotments on form 1-B to notify of change in address of allottee. . . . . . . . . . . | | 74 | | |
| Liberty Loan Bond . . . . . . . . . . . . | | 27<br>69 | II | |
| May be made by officers, enlisted men and field clerks . . . . . . . . . . . . . | 164 | | | |
| Not effected by sentence of court-martial imposing forfeiture of pay. . . . . . . | | 45<br>47 | | |
| Pay, non-receipt of, by dependents at home. . . . . . . . . . . . . . . . . . | | 29 | | |
| Procedure in order to secure prompt payment of. . . . . . . . . . . . . . . . . | 137 | | I | |
| **Allowance —** | | | | |
| Clothing . . . . . . . . . . . . . . . . . | 25 | | V | 1-8 |
| Additional to tables of organization. . . . | 92 | | | |
| Increase for purchase of music for bands. | | 53 | | |
| Of bicycles. . . . . . . . . . . . . . . . | 92 | | | |
| Of fuel. . . . . . . . . . . . . . . . . . | 8 | | III | |
| To be paid under conditions, and at the rate specified herein. . . . . . . . . . | 132 | | I | 4 |
| **Ambulances —** | | | | |
| Gas heaters in . . . . . . . . . . . . . . | 2 | | II | |
| Danger of gas heaters in . . . . . . . . | 2 | | II | |
| Ventilation of. . . . . . . . . . . . . . | 2 | | II | |
| Ford with inclosed bodies. . . . . . . . . | 2 | | II | |
| **American Expeditionary Forces, Headquarters —** | | | | |
| Official title of . . . . . . . . . . . . . | 11 | | I | |
| **American —** | | | | |
| Liaison officers at French Regional Headquarters. . . . . . . . . . . . . . . . . | 32 | | III | 3 |
| Word to be written in full addressing mail or telegraphic communications to units and individuals in American E. F. | 155 | | III | |
| **American Military Mission —** | | | | |
| Issuance of passes to Red Cross and Y. M. C. A. . . . . . . . . . . . . . . . | 33 | | V | |
| **American Red Cross —** | | | | |
| Assignments to Graves Registration Service. . . . . . . . . . . . . . . . . . . | 101 | | | |
| With the land and naval forces. . . . . | 139 | | | |

| PURPORTS | G. O. | Bul. | Sec. | Par. |
|---|---|---|---|---|
| **Animals —** | | | | |
| Care and treatment of. . . . . . . . . . | 65 | | | |
| Daily forage rations. . . . . . . . . . . | 174 | | II | |
| Designation of, before being sent to Veterinary Hospital. . . . . . . . . . . . . | 33 | | III | |
| Disposition of. . . . . . . . . . . . . . | | 19 | | |
| Issue, evacuation, accountability and inspection of . . . . . . . . . . . . . . | 122 | | II | 5-8 |
| Numbers reduced . . . . . . . . . . . . | 92 | | | |
| Rations for . . . . . . . . . . . . . . . . | 34 | | III | |
| **Appointments and Promotions, Officers —** | | | | |
| Recommendations. . . . . . . . . . . . | 24 | | I | 1-3 |
| **Arctic Overshoes —** | | | | |
| The issue of as authorized, will cease when the supply now on hand is exhausted. . . . . . . . . . . . . . . . . | 142 | | | |
| **Area Stores —** | | | | |
| To be issued regiments . . . . . . . . . | 12 | | I | 3 |
| **Arm and Ordnance Equipment —** | | | | |
| How issued, accountability for same . . . | 150 | | III | |
| **Army —** | | | | |
| Abolishing distinction between R. A., N. A., N. G. and Reserve Corps. . . . | 144 | | I | |
| Engineer Troops, general duties, control, assigned to certain areas. . . . . . . . | | 65 | | |
| Intelligence school, establishment of. . . | 130 | | VII | |
| Organization of 2nd. . . . . . . . . . . | 175 | | | |
| Radio Section, regulations governing same. | 152 | | III | |
| Serial number. . . . . . . . . . . . . . . | 130 | | VIII | |
| **Army Candidates' School —** | | | | |
| Establishment of . . . . . . . . . . . . | 32 | | I | |
| Transfer of candidates. . . . . . . . . . | 45 | | IV | |
| **Army Field Clerks —** | | | | |
| Entitled to commutation of quarters, heat, and light on account dependents. . . . | 78 | | | |
| **Army Serial Number —** | | | | |
| Of soldiers . . . . . . . . . . . . . . . . | 130 | 49 | | |
| **Army Repair Shops**. . . . . . . . . . . . | 43 | | V | |
| **Articles for Members of A. E. F. —** | | | | |
| Shipment of. . . . . . . . . . . . . . . . | | 24 | | |
| Violations of instructions . . . . . . . . | | 43 | II | |
| **Articles of Clothing —** | | | | |
| To whom may be sold . . . . . . . . . . | 104 | | | |
| Withdrawn from sale to enlisted men . . | 37 | | I | 1 |
| **Articles of Subsistence —** | | | | |
| Required for sick in hospitals, sale of such discontinued by sales Commissary . . . | | 67 | | |
| **Auto-Chirs —** | | | | |
| Autonomes . . . . . . . . . . . . . . . . | 70 | | II | |
| **Automobile Headlights —** | | | | |
| Use of, during airplane warning . . . . . | 23 | | I | |
| **Autopsy —** | | | | |
| When needed authority to be secured. . . | 10 | | I | |
| **Aviators —** | | | | |
| Official credit to . . . . . . . . . . . . | | 76 | | |

| PURPORTS | G-O. | Bul. | Sec. | Par. |
|---|---|---|---|---|
| **Awards** — | | | | |
| For gallantry in action and meritorious service | | 25 | | |
| **Axes** — | | | | |
| Sharpening of | 21 | | III | 6 |
| **Badge** — | | | | |
| None other than authorized under G. O. 155 W. D. Wash. to be worn | | 70 | | |
| **Bags** — | | | | |
| Jute, use of, in shipment of bread | 20 | | I | 1-5 |
| **Bakeries** — | | | | |
| Fuel Allowance | 117 | | I | 8 |
| **Bakers** | 47 | | | |
| **Band** — | | | | |
| Using of members as stretchers bearers to be discontinued, see exception | 139 | | III | |
| **Bands** — | | | | |
| Allowance increased | | 73 | | |
| Initial allowance for new | | 73 | | |
| Purchase of music for | | 73 | | |
| **Base Censor** — | | | | |
| Duties of | 146 | | II | (c) |
| **Base Training Schools** | 47 | | | |
| **Bathing Units** — | | | | |
| Personnel of | 60 | | III | |
| **Baths** — | | | | |
| Location and maintenance of, by Town Majors | 18 | | | 7 |
| Fuel Allowance | 117 | | I | 10-13 |
| **Battle** — | | | | |
| Training for | 4 | | | 1 |
| **Bed Sacks** — | | | | |
| Material for filling | | 36 | | |
| **Bedding** — | | | | |
| Issue of hay as | 41 | | VII | |
| **Beef** — | | | | |
| Tallow | | 5 | | |
| **Beehives** — | | | | |
| Destruction of, by soldiers | 42 | | III | |
| **Beneficiary Designation Cards** — | | | | |
| Form 380 A. G. O. no longer required | 174 | | IV | |
| **Billeting and Quartering of Troops in France** — | | | | |
| Requisitions | 18 | | | |
| Rules governing and general provisions | 18 | | | |
| Things not liable to be requisitioned | 18 | | | 16 |
| Things not liable to be requisitioned | 48 | | III | |
| Renting, Requisition and Claim Service | 50 | | IV | |
| Renting and quartering | 60 | | II | |
| **Billets** — | | | | |
| Fires occurring in, prevention of | 43 | | I | |
| **Bills** — | | | | |
| Monthly for telephone, etc., from French Gov't | | 67 | | |
| Bivouac | | 2 | | |

| PURPORTS | G. O. | Bul- | Sec. | Par. |
|---|---|---|---|---|
| **Blank Forms and Books —** | | | | |
| Distribution of, Instructions re same . . . | | 71 | | |
| **Blankets, Dirty —** | | | | |
| Prevalence of . . . . . . . . . . . . . . . | 4 | | | 1-3 |
| **Blue Envelopes —** | | | | |
| How issued. . . . . . . . . . . . . . . . | 146 | | | |
| **Board of Allied Supply —** | | | | |
| Military appointment of . . . . . . . . . | 100 | | III | |
| **Board of Contracts and Adjustments —** | | | | |
| Duties of . . . . . . . . . . . . . . . . | 29 | | II | 1-3 |
| **Board of Officers —** | | | | |
| To make recommendations as to efficiency. | 45 | | | |
| **Boards —** | | | | |
| Disability . . . . . . . . . . . . . . . . | 41 | | I | 1-8 |
| **Bombs —** | | | | |
| Shells and grenades unexploded, danger of handling . . . . . . . . . . . . . . . | | 27 | | |
| **Bonds —** | | | | |
| Liberty Loan — 3rd . . . . . . . . . . . | 59 | | | |
| Liberty Loan — 4th. . . . . . . . . . . | 164 | | | |
| National defense . . . . . . . . . . . . . | | 27 | III | |
| Second alphabetical list . . . . . . . . . | 117 | | IV | |
| Sale, transfer prohibited . . . . . . . . . | | 69 | | |
| **Book —** | | | | |
| Pay record, adopting of an individual. . . | 126 | | I | |
| **Boxes —** | | | | |
| Wooden, salvage of . . . . . . . . . . . | 10 | | III | 1 |
| **Bread —** | | | | |
| Allowance, increase of, to troops of front. | 33 | | VI | |
| Bread cards . . . . . . . . . . . . . . . | 54 | | | |
| Handling of. . . . . . . . . . . . . . . . | | 21 | | |
| Shipping of, in jute bags by bakeries. . . | 20 | | II | |
| Unleavened " Matzos " . . . . . . . . . . | 28 | | III | |
| Waste of, prevention. . . . . . . . . . . | 20 | | III | |
| **Building Materials —** | | | | |
| Toll on, called Octroi Dues — how paid . | | 66 | | |
| **Bulletins —** | | | | |
| Amendments and revocations of bulletins, G. H. Q., 1918 : | | | | |
| No 3, 1917, revoked by. . . . . . . . | | 40 | I | |
| No 4, Sec. III, 1917, amended by. . . . | | 60 | I | |
| No 6, Sec. I, 1918, revoked by . . . . . | | 15 | I | |
| No 8, 1918, modified by . . . . . . . . | | 58 | II | |
| No 10, 1917, amended by. . . . . . . . | 18 | | | |
| | 48 | | III | |
| | 50 | | IV | |
| No 10, 1918, if conflicting revoked by. . | | 26 | | 9 |
| No 15, 1917, amended by. . . . . . . . | 5 | | | |
| | 25 | | | |
| No 16, 1917, amended by. . . . . . . . | 5 | | | |
| | 25 | | | |
| No 19, 1917, supplemented by . . . . . | 44 | | | |
| No 20, Par. 3, 1917, revoked by. . . . | | 20 | I | |
| No 23, 1918, partly revoked by. . . . . | | 42 | II | |
| No 25, 1918, supplemented by . . . . . | | 56 | I | |

| PURPORTS | G. O. | Bul. | Sec. | Par. |
|---|---|---|---|---|
| **Bulletins** (Con'd) — | | | | 5 |
| No 30, 1918, partly revoked by | 91 | | | |
| No 35, Sec. II, par. 3-A, 1918, amended by | | 48 | I | |
| No 60, Sec. I, 1918, revoked by | | 66 | II | |
| **Bulletins and General Orders** — | | | | |
| Distribution of | | 15 | | |
| **Bureau** — | | | | |
| Burial Department | 50 | | | |
| Personnel | 94 | | I | |
| Of Naturalization | | 68 | | |
| Of reciprocal supply | 152 | | | |
| Of War Risk Insurance | | 74 | | |
| **Burial Reports** — | | | | |
| Rules for | 50 | | I | |
| | | 41 | I | |
| **Burials** — | | | | |
| Of Jewish Soldiers | 122 | | II | |
| Regulation for | 30-50 | | | |
| Reports of | 89 | | | |
| **Cable Censorship Regulations** | 146 | | | |
| **Cablegrams** — | | | | |
| E. F. M. | | 40 | | |
| **Calls** — | | | | |
| Long Distance | | 48 | I | |
| **Camouflage** | | 37 | | |
| | | 17 | II | |
| **Camp** | | 2 | | |
| **Camp Sites** — | | | | |
| Granted thru French Military Mission | 18 | | | 66 |
| | 60 | | | |
| **Candidates Ordered To Army Candidates School** — | | | | |
| Transfer of | 45 | | | |
| Sleeve stripes, how worn | 121 | | III | 4 |
| **Candidates School, Army** — | | | | |
| Eligibility for | 32 | | | |
| Transfer of Candidates | 45 | | V | |
| Minimum age limit amended | 121 | | III | 1 |
| **Candles** — | | | | |
| Increase in allowance | 62 | | | |
| Lighting of, forbidden in barns or outhouses | 18 | | | 15-i |
| **Cantonments** | | 2 | | |
| **Caps** — | | | | |
| Model to be worn by Officers | 149 | | I | |
| Overseas adopted for | 7 | | I | 6 |
| Overseas, insignia of rank to be worn | 64 | | IX | |
| Service may be worn | 7 | | I | 9 |
| **Casual Officers and Soldiers** — | | | | |
| Veterinary | 122 | | II | 9 |
| Disposition of | 68 | | | 3 |
| Casual Reports | 100 | | | |
| Form of casual reports | 170 | | | |
| Casualty reports, telegraphing of, code equivalents | | 46 | | |

| PURPORTS | G.O. | Bul. | Sec | Par. |
|---|---|---|---|---|
| **Cemeteries —** | | | | |
| Acquisition of land for, by Graves Registration Service. . . . . . . . . . . . . | | 41 | | |
| **Censor Stamps —** | | | | |
| How issued, not transferable. . . . . . . | 146 | | | |
| Officers using same to exercise care . . . | | 75 | | |
| Re Provost Marshal Stations. . . . . . . | 6 | | | 17 |
| **Censhorhip —** | | | | |
| Officers will require name of language to be noted on letter. . . . . . . . . . . | 14 | | III | |
| Of photographs. . . . . . . . . . . . . | 15 | | | |
| Stamps . . . . . . . . . . . . . . . . . . | 34 | | VII | |
| Regulations in full — (revoking G. O. 13, 1917, and others) . . . . . . . . . . . | 146 | | | |
| **Central Records Office —** | | | | |
| See. . . . . . . . . . . . . . . . . . . . | 89<br>100 | | | |
| Re Service records of soldiers . . . . . . | 165 | | II | |
| **Certificates —** | | | | |
| Bread and sugar. . . . . . . . . . . . . | 54 | | | |
| **Challes-les-Eaux —** | | | | |
| Leave centre for soldiers. . . . . . . . . | 38 | | | 1-3 |
| **Chambery —** | | | | |
| Leave centre for soldiers. . . . . . . . . | 38 | | | 1-3 |
| **Change —** | | | | |
| In designation of Sanitary Squads . . . . | 125<br>153 | | | |
| Of duties and status of Officers, reporting . . . . . . . . . . . . . . . . . . | | 49 | I | |
| Of Name, upon application of naturalization. . . . . . . . . . . . . . . . . . | | 77 | | |
| **Chaplains —** | | | | |
| Duties of, in the divisions. . . . . . . . | | 72 | | |
| Letters of sympathy. . . . . . . . . . . | 89 | | | |
| Marking Jewish Soldiers' Graves. . . . . | 122 | | III | 2 |
| Upon arrival for duty with A. E. F., report to A. G. O. . . . . . . . . . . . . | 77 | | IV | |
| When relieved from duty with divisions, to be sent to School at Le Mans for reclassification . . . . . . . . . . . . . | 163 | | II | |
| **Chaplains Office —** | | | | |
| Functions of . . . . . . . . . . . . . . . | 66 | | VIII | |
| **Chauffeurs —** | | | | |
| Authorized speed for. . . . . . . . . . . | 80 | | | |
| Ambulance . . . . . . . . . . . . . . . . | 2 | | II | 5 |
| Commutation allowances . . . . . . . . . | 167 | | | |
| Report of Accidents. . . . . . . . . . . . | 11 | | III | 3 |
| School for. . . . . . . . . . . . . . . . . | 47 | | | |
| **Checks Duplicate —** | | | | |
| When issued by Disbursing Officers. . . | | 55 | | |
| **Chemical Warfare Service —** | | | | |
| Formerly called Gas Corps . . . . . . . | 105 | | VII | |
| **Chevrons** . . . . . . . . . . . . . . . . . . | | 18 | I | |
| Computation of time . . . . . . . . . . | 104 | | III | |
| Wound and War Service. . . . . . . . . | 26 | | I | |

| PURPORTS | G. O. | Bul. | Sec. | Par. |
|---|---|---|---|---|
| **Chevrons** (Cont'd) — | | | | |
| Wearing of. . . . . . . . . . . . . . . . | 33<br>73<br>75 | | IV<br>III<br>II | |
| **Chief Consultant** — | | | | |
| Medical Services, A. E. F. announced. . . | 88 | | | |
| Surgical Services A. E. F.. . . . . . . . . | 88 | | | |
| **Chief** — | | | | |
| Liaison Officer . . . . . . . . . . . . . | 28 | | | |
| Of Remount Service. . . . . . . . . . . | 122 | | II | 3 |
| Of Tank Corps, duties . . . . . . . . . . | 121 | | I | |
| Of Veterinary Service . . . . . . . . . . | 122 | | II | |
| Surgeon announced . . . . . . . . . . . | 66 | | III | |
| **Chinese Laborers** — | | | | |
| Rations to be issued to . . . . . . . . . | 1 | | IV | |
| **Citation** — | | | | |
| To be furnished with each award of D. S. C. or M. of H.. . . . . . . . . . . | 144 | | II | |
| **Civilian Employees** — | | | | |
| Local engagement of . . . . . . . . . | 32 | | | |
| Request for employment, male or female, how to be submitted. . . . . . . . . . | 131 | | V | |
| Ration charges when admitted to hospitals. | 53 | | III | |
| **Class "D"** — | | | | |
| Regulations relating to Officers and enlisted men classified in hospitals. . . . | 161 | | I | |
| **Claims** . . . . . . . . . . . . . . . . . . . . . | 50<br>78 | | IV<br>IV | |
| Of soldiers for private property, lost etc. | | 52 | | |
| Re damages. . . . . . . . . . . . . . . . | 18<br>78 | | IV | |
| **Classification and Methods of Supply**. . . . | 44 | | | 23-24 |
| **Clean Clothing** — | | | | |
| Issue of. . . . . . . . . . . . . . . . . . | 13 | | I | 3 |
| **Clothes Driers** — | | | | |
| Plans for. . . . . . . . . . . . . . . . . | 11 | | IV | 8 |
| **Clothing Allowance**. . . . . . . . . . . . . | 25 | | V | |
| **Coal and Wood** — | | | | |
| Allowance. . . . . . . . . . . . . . . . . | 117 | | I | 3 |
| Prices. . . . . . . . . . . . . . . . . . . | 117 | | I | 15 |
| **Coke and Charcoal** — | | | | |
| Allowance. . . . . . . . . . . . . . . . . | 117 | | I | 3 |
| Careful use of. . . . . . . . . . . . . . . | 20 | | I | |
| Not to be used in unventilated quarters. . | 117 | | I | 4 |
| When issued. . . . . . . . . . . . . . . . | 117 | | I | 4 |
| **Collisions** — | | | | |
| Careless. . . . . . . . . . . . . . . . . . | 11 | | III | 1 |
| Claims for damages . . . . . . . . . . . | 11 | | III | 2 |
| **Commutations** — | | | | |
| Allowances for Nurses, Telephone operators etc. . . . . . . . . . . . . . . . . | 132 | | | |
| Of quarters, heat and light for Field Clerks . . . . . . . . . . . . . . . . . | | 78 | | |
| Of rations, to enlisted chauffeurs, drivers, etc. . . . . . . . . . . . . . . . . | 167 | | III | |

| PURPORTS | G. O. | Bul. | Sec. | Par. |
|---|---|---|---|---|
| **Commutations** (Cont'd) — | | | | |
| Of enlisted men ordered on leave . . . . | 38 | | I | 3 |
| Payments to be made on certificates of Officers. . . . . . . . . . . . . . . | 63 | | IV | |
| Rates for sick enlisted men. . . . . . . . | 30 | | VIII | |
| To enlisted men. . . . . . . . . . . . . . | 32 | | II | |
| To Intelligence Police. . . . . . . . . . . | 3 | | II | |
| To Officers and Soldiers of the French Army, serving with the A. E. F . . . . | 132 | | | |
| To Civilian Employees . . . . . . . . . | 132<br>53 | | | |
| **Confinement** — | | | | |
| In disciplinary Barracks. . . . . . . . . . | 155 | | IV | |
| **Conservation of Food**. . . . . . . . . . . . | 70 | | | 2 |
| **Construction and Repair Work** — | | | | |
| Engineer and Q. M. table . . . . . . . . | 32 | | V | |
| **Construction and Forestry** — | | | | |
| To conduct all negotiations for standing timber, exceeding 5000 francs in value. . . . . . . . . . . . . . . . . . | 159 | | III | |
| **Contracts and Adjustments** — | | | | |
| Board of . . . . . . . . . . . . . . . . | 29 | | | |
| **Control of Prisoners of War** — | | | | |
| Treatment of etc. . . . . . . . . . . . . . | 159 | | | |
| **Cookers** — | | | | |
| Field, Plans for. . . . . . . . . . . . . . | 11 | | IV | 8 |
| **Cooks and Mess Sergeants** — | | | | |
| Allowance at Army Schools and Corps Schools. . . . . . . . . . . . . . . . . | 105 | | IV | 8 |
| **Cooks** — | | | | |
| School for . . . . . . . . . . . . . . . . | 47 | | | |
| **Correspondence** — | | | | |
| With B. E. F. Official. . . . . . . . . . . | 33 | | III | 1-2 |
| With French Departments. . . . . . . . | 8 | | I | |
| With regard to method of dating. . . . . | 25 | | III | |
| **Correspondents** — | | | | |
| Accredited . . . . . . . . . . . . . . . . | 96 | | | |
| Permission to take photographs. . . . . . | 15 | | | |
| Sales of subsistence to. . . . . . . . . . | 159 | | | |
| Visiting. . . . . . . . . . . . . . . . . . . | 96 | | | |
| **Courses** — | | | | |
| Gas. . . . . . . . . . . . . . . . . . . . . | 35 | | | 10 |
| Reports upon completion of course. . . . | 35 | | | 14 |
| **Court-Martial** — | | | | |
| Empowering appointment of General. . . | 53<br>118<br>137 | | | |
| Holding of, for accidents with motor vehicles. . . . . . . . . . . . . . . . . | 11 | | III | 3 |
| New modes of punishment by . . . . . . | 56 | | I | |
| Portion of pay forfeiture through . . . . | 16 | | II | |
| Witnesses, attendance of, promptness of . | 87 | | | |
| **Damages** — | | | | |
| Indemnity for, caused by A. E. F. . . . . | 78 | | IV | |
| To Civilian property. . . . . . . . . . . | | 24 | II | |

| PURPORTS | G. O. | Bul. | Sec. | Par. |
|---|---|---|---|---|
| **Dead** — | | | | |
| Internment of enemy's | 50 | | I | |
| **Deaths** — | | | | |
| Notifications of families | 89 | | | |
| Report of, etc. | 40 | | | |
| Report of Officers, soldiers, or civilians serving with the A. E. F. | 170 | | II | |
| Re new Forms to be used for reports | 178 | | II | |
| **Deceased** — | | | | |
| Enlisted | 10 | | | |
| Notification of, to families | 89 | | | |
| Officers | 10 | | | |
| **Decisions** — | | | | |
| Advance, request of, by Ass't. Compt. of Treasury | | 16 | II | |
| **Decorations and Insignia** — | | | | |
| Foreign, wearing permitted | | 24 | II | |
| **Degassing Unit** — | | | | |
| Organization and equipment of | 144 | | VIII | |
| **Dental Attendance** — | | | | |
| Personnel and material for combat division | 99 | | I | |
| **Designation of Animals** — | | | | |
| Before being sent to Veterinary hospitals | 30 | | III | |
| **Detachment** — | | | | |
| Mail | 155 | | II | |
| **Director** — | | | | |
| Of General Medicine | 38 | | IV | |
| Of Gas School | 35 | | | |
| Of professionnal services | 88 | | | |
| Of Roentgenology | 36 | | III | |
| **Disability Boards** | 41 | | I | 1-8 |
| **Disbursing Officers** — | | | | |
| May issue duplicate checks to replace lost, stolen, or destroyed | | 55 | | |
| Request of advance decision of Ass't. Compt | | 16 | | |
| To furnish specimen signatures | 37 | | IV | |
| Under payment of moneys | | 11 | | |
| **Discharge** — | | | | |
| Dishonorable | 56<br>78<br>155 | | <br>I<br>IV | <br>1<br> |
| **Discipline** | 56<br>78<br>87 | | | |
| **Diseases** — | | | | |
| Epidemic among troops | 32 | | | |
| Epidemic among Civilian Population | | 28 | | |
| Trench foot | 11 | | IV | |
| Venereal | 6 | | I | 11 |
| **Disinfectors** — | | | | |
| Laundry and Bathing Units : | | | | |
| Fuel, Allowance | 117 | | I | 9-13 |
| Personnel of | 60 | | III | |

| PURPORTS | G. O. | Bul. | Sec. | Par. |
|---|---|---|---|---|
| **Distinguished Service Cross** . . . . . . . . | 26 | | | |
| | 48 | | V | |
| | 144 | | II | |
| | | 56 | | |
| **Distinguished Service Medal** . . . . . . . . | 26 | | | |
| **Distribution** — | | | | |
| Of General Orders and Bulletins . . . . | | 15 | | |
| **Doctors' Caps and Aprons** — | | | | |
| To be laundered by Q. M. C. | 13 | | I | 1 |
| **Dogs** — | | | | |
| Not to be in possession of members of A. E. F., except when officially authorized. . . . . . . . . . . . . . . . . . . | 167 | | II | |
| **Drivers** — | | | | |
| Of motor vehicles, commutation allowance. | 167 | | III | |
| Report, required in case of damages. . . . | 11 | | III | 3 |
| Responsibility for accessories . . . . . . . | | 73 | | |
| School for. . . . . . . . . . . . . . . . . . | 47 | | | |
| **Dubbin**. . . . . . . . . . . . . . . . . . . . | | 48 | II | |
| **Duties** — | | | | |
| Of Chaplains in the divisions . . . . . . | | 72 | | |
| Of Military Police Corps . . . . . . . . | 158 | | III | |
| Of Statistical Division. . . . . . . . . . . | 174 | | III | |
| **Ear, Nose and Throat Surgery** — | | | | |
| Senior Consultant. . . . . . . . . . . | 88 | | | |
| **Effects Depot** — | | | | |
| Establishment of . . . . . . . . . . . . . | 40 | | | 1-11 |
| **Efficiency of Officers** — | | | | |
| Investigations. . . . . . . . . . . . . . . | 45 | | | |
| **Effects Section** — | | | | |
| Receipt of burial report of. . . . . . . . | 30 | | IV | 7 (f) |
| **Electric Light Installation** . . . . . . . . . | 33 | | I | 1- |
| **Electric Machinery**. . . . . . . . . . . . . . | 33 | | | 3 |
| **Employees, Civilians** — | | | | |
| Rations when admitted to hospitals . . . | 53 | | III | |
| Re local hire of. . . . . . . . . . . . . . | 32 | | III | 2 |
| Request for male and female, how to be submitted. . . . . . . . . . . . . . . | 131 | | V | |
| **England** — | | | | |
| Leave of troops in. . . . . . . . . . . . | 6 | | | 22 |
| **Envelopes Blue** — | | | | |
| How issued. . . . . . . . . . . . . . . | 146 | | | |
| **Equipment of Enlisted Men** — | | | | |
| Transferred with men from organization. | 25 | | V | 2 |
| **Equipment** — | | | | |
| Personnel, when carried on leave . . . . | 6 | | | |
| Railways, damage to, record of. . . . . . | 66 | | VII | |
| Surplus, disposition of. . . . . . . . . . | 10 | | II | |
| **Exhaust Gas Heaters** — | | | | |
| Ford Ambulances, equipment with, dangerous . . . . . . . . . . . . . . . . . | 2 | | II | |
| **Evacuating and Sorting Stations**. . . . . | 44 | | | 22 |
| **Evacuation of Animals**. . . . . . . . . . . | 122 | | II | 6 |
| **Fagots** — | | | | |
| Bundling of. . . . . . . . . . . . . . . . | 21 | | III | 8 |

| PURPORTS | G. O. | Bul. | Sec. | Par. |
|---|---|---|---|---|
| **Fatigue Clothing —** | | | | |
| Issue to Officers . . . . . . . . . . . . . | 7 | | I | 3 |
| **Feed Bags —** | | | | |
| Salvage of . . . . . . . . . . . . . . . . | 10 | | III | 1 |
| **Feet —** | | | | |
| Hygiene of. . . . . . . . . . . . . . . | 11 | | IV | 4 (e-h) |
| **Field Censorship Regulations** . . . . . . . . | 146 | | | |
| **Field Clerks —** | | | | |
| Commutations of quarters, heat and light. | | 78 | | |
| Leave of absence . . . . . . . . . . . . | 6 | | | |
| Minimum pay and allowance. . . . . . . | 119 | | | |
| **Field Hospitals —** | | | | |
| To be disinfected by Medical Corps. . . . | | 12 | | |
| **Field Ranges** . . . . . . . . . . . . . . . . | | 14 | I | |
| **Flour Bags —** | | | | |
| Salvage of. . . . . . . . . . . . . . . . | 10 | | III | 1 |
| **Food Supplies —** | | | | |
| Purchase of, by messes and individuals of A. E. F. . . . . . . . . . . . . . . . | | 33 | | |
| **Foot Powders —** | | | | |
| Issue by Medical Department . . . . . . | 11 | | IV | 9 |
| **Footwear —** | | | | |
| Drying of. . . . . . . . . . . . . . . . . | 11 | | IV | 4 (d) |
| Supply of. . . . . . . . . . . . . . . | 58 | | V | |
| **Forage —** | | | | |
| Ration prescribed . . . . . . . . . . . . . | 14 | | IV | |
| | 34 | | III | |
| | 66 | | IV | 3 |
| Prescribed for animals in A. E. F . . . . | 174<br>79 | | III | |
| **Form No.** 218 — | | | | |
| To be accomplished by Officers . . . . . | 62 | | III | |
| **Forms —** | | | | |
| Distribution of Blank, and Book . . . . . | | 71 | | |
| Duty status, of officers and enlisted men. | 170 | | II | |
| Prescribed for naturalization of aliens with A. E. F. | 151 | | | |
| **Free Duty into Italy on War Material and Equipment for Troops** . . . . . . . . . . | | 17 | III | |
| **Freight Carmarking —** | | | | |
| New posters for . . . . . . . . . . . . | 17 | | II | 2 |
| **Freight Cars Loaded —** | | | | |
| Notifying car record office of location of . | 17 | | I | 4-5 |
| Tracing of . . . . . . . . . . . . . . . . . | 17 | | I | 6 |
| **Freight Transportation —** | | | | |
| Change in carmarking method . . . . . . | 17 | | II | 2 |
| **French Officers and Soldiers Attached to the A. E. F. —** | | | | |
| Rate of pay . . . . . . . . . . . . . . . | 50 | | II | |
| **French Telephones and Telegraphs —** | | | | |
| System, Use of . . . . . . . . . . . . . | | 28 | | |
| **Fuel —** | | | | |
| Allowance, Conservation of, Distribution of. . . . . . . . . . . . . . . . . . . . | 117 | | I | [illegible] |
| French unauthorized use of, forbidden . . | 18 | | II | |

| PURPORTS | G. O. | Bul. | Sec. | Par. |
|---|---|---|---|---|
| **Fuel** (Cont'd) — | | | | |
| Increased allowance | 8 | | III | |
| Method of distribution | 44 | | | 25 |
| **Funds** — | | | | |
| Custodians of, exempted from billets providing | 18 | | | 25 |
| Discharged officers of, rules for | 127 | | | 5 |
| Public | 117 | | I | 3-16 |
| Surplus exchange and mess, how and when invested in Liberty Loans — | 164 | | | 6 |
| **Furloughs** | 6 | | | |
| **Garden Service** | 34 | | II | |
| **Garrison Ration** — | | | | |
| Component articles and substitutive quantities | 176 | | II | |
| **Gas Corps** | 79 | | | |
| **Gas Defense Schools** | 79 | | II | |
| **Gas Heaters** — | | | | |
| In ambulances | 2 | | II | |
| **Gas Masks** — | | | | |
| Classification of | 28 | | II | 1-2 |
| **Gas Mustard** — | | | | |
| Tar paper a protection against | | 75 | | |
| **Gas Units** — | | | | |
| Organization and equipment of | 144 | | VIII | |
| **Gas Warfare** | 79 | | I | 1 |
| **Gasoline and Oils** — | | | | |
| Economy in use of | 19 | | I | 1 |
| Requisitioning of | 48-66 | | | |
| **Gas School** — | | | | |
| Organization of | 35 | | | 10 |
| **Gas Service : Chief of, First Army Corps** — | | | | |
| Appointee | 9 | | | |
| **General Headquarters, A. E. F.** — | | | | |
| Designation | 11 | | | |
| Distribution of Staff Duties | 31 | | | |
| **General Medical Inspector** — | | | | |
| Officers empowered to appoint | 58<br>118 | | <br>III | |
| Announced | 29 | | IV | |
| **General Orders and Bulletins** — | | | | |
| Distribution of | | 15 | | |
| **General Orders** — | | | | |
| Revocations, amendments, modifications, etc. | | | | |
| No 2, 1917, Amended by | 100 | | I | 7 |
| No 2, Sec. II, 1917, Amplified by | 31 | | | |
| No 3, Sec. II, 1918, Amended by | 38 | | III | |
| No 6, 1918, if conflicting revoked | 38 | | I | 4 |
| No 6, Par. 9, 1918, amended by | 135 | | II | |
| No 6, Par. 12, 1918, amended by | 15 | | III | |
| No 6, Par. 21, 1918, amended by | 29 | | III | |
| No 7, 1917, attention directed to | 42 | | III | |
| No 7, Par. 8, 1918, revoked by | 148 | | I | |
| No 8, 1917, revoked by | 31 | | | |
| No 9, 1917, Sec. I, revoked by | 199 | | V | |

| PURPORTS | G. O. | Bul. | Sec. | Par. |
|---|---|---|---|---|
| **General Orders** (Cont'd) — | | | | |
| No 10, Sec. III, Pars. 7-8-9-10-10a, 1918, amended by. . . . . . . . . . . . . | 73 | | II | 1 |
| No 10, Par. 3 (sub. 7), 1918, revoked by. | 28 | | II | |
| No 11, Sec. III, Par. 4, 1918, amended by. | 50 | | IV | 2 E (5) |
| No 12, Par. 1, 1918, revoked by. . . . . | 49 | | | |
| No 13, 1917, revoked by . . . . . . . . | 146 | | | |
| No 14, Sec. IV, 1918, modified by. . . . | 34 | | III | 2 |
| No 15, Sec. 1. 1918, amended by . . . . | 78 | | III | |
| No 15, 1918, amended by. . . . . . . . | 121 | | VII | |
| No 15, Sec. I, 1918, if conflicting revoked by . . . . . . . . . . . . . . . . . . | 146 | | | |
| No 16, 1917, supplemented by . . . . . | 10 | | III | |
| No 16, Par. 10, 1918, revoked by. . . . | 42 | | II | |
| No 16, Sec. IV, 1918, revoked by. . . . | 58 | | IV | |
| No 18, Sec. 2, 1917, amended by. . . . . | 84 | | I | |
| No 18, 1918, amended by. . . . . . . . | 48 | | III | |
| No 18, Pars. 51-52, 1918, amended by. . | 50 | | IV | 2 E (4) |
| No 19, Par. I, 1917, amended by. . . . | 100 | | I | 7 |
| No 19, Sec. IV, Par. 2, 1917, revoked by. | 170 | | V | |
| No 19, Sec. II, 1918, amended by . . . . | 50 | | II | |
| | 62 | | I | |
| No 21, 1917, if conflicting, revoked by . | 30 | | V | |
| No 21, Sec. VI, 1917, if conflicting, revoked by . . . . . . . . . . . . . . . | 91 | | II | |
| No 21, Sec. I, 1918, revoked by . . . . | 199 | | I | |
| No 22, Sec. III, 1917, modified by . . . | 154 | | III | |
| No 22, Par. 1, 1917, revoked by . . . . | 15 | | I | |
| No 22, 1918, revoked by . . . . . . . . | 117 | | I | 1 |
| No 23, Sec. I, Par. 5, 1917, supplemented by . . . . . . . . . . . . . . . . . . | 105 | | I | 1 |
| No 23, Sec. IV, Par. 2, 1917, supplemented by. . . . . . . . . . . . . . | 46 | | | |
| No 23, Sec. III, 1918, revoked by . . . | 68 | | I | 1 |
| No 24, 1918, revoked by . . . . . . . . | 144 | | I | 2 |
| No 25, Sec. III, 1917, amended by . . . | 63 | | I | |
| No 25, Sec. II, Par. 1, 1918, rescinded by. | 185 | | III | |
| No 26, 1918, amended by. . . . . . . . | 30 | | II | |
| No 26, Par. 1- (c), 1918, amended by . . | 33 | | IV | |
| No 27, 1917, re burials, revoked by. . . | 30 | | V | |
| No 29, 1917, if conflicting, revoked by . | 180 | | III | 20 |
| No 29, Sec. I, Par. 2, 1918, amended by. | 41 | | IV | |
| No 30, 1917, if conflicting, revoked by . | 180 | | III | 20 |
| No 30, Sec. II, 1918, revoked by. . . . | 89 | | II | |
| No 30, Sec. IV, Par. 2-N, 1918, amended by . . . . . . . . . . . . . . . . . . | 53 | | II | |
| No 30, Sec. IV, Par. 7-N, 1918, if conflicting, revoked by . . . . . . . . . | 91 | | II | |
| No 30. Sec. IV, Par. 7-F, 1918, amended by . . . . . . . . . . . . . . . . . | 100 | | | 14 |
| | 48 | | II | |
| | 64 | | I | |
| No 31, 1918, amended by. . . . . . . . | 81 | | I | 6 |
| | 111 | | XI | 15 |
| | 114 | | I | 1 |

| PURPORTS | G. O. | Bul. | Sec. | Par. |
|---|---|---|---|---|
| **General Orders** (Cont'd) — | | | | |
| | 121 | | I | 7 |
| | 131 | | II | E |
| No 31, 1918, amended by (Cont'd). . . . | 139 | | II | 1 |
| | 144 | | IV | 1 |
| | 180 | | II | |
| No 32, 1918, amended by. . . . . . . . | 45 | | III | 2 |
| No 32, Sec. I, Pars. 2-3, 1918, amended by. . . . . . . . . . . . . . . . . | 121 | | III | 1-2 |
| No 32, Sec. I, 1918, amended by . . . . | 95 | | | |
| No 32, Sec. I, Par. 8, 1918, amended by. | 171 | | II | |
| No 32, Sec. III, Par. 1, 1918, revoked by. | 152 | | I | 3 |
| No 32, Sec. III, Par. 1-(a), 1918, amended by . . . . . . . . . . . . . . . | 182 | | V | |
| No 33, Sec, IV, 1918, revoked by. . . . | 147 | | III | |
| No 34, Sec. VI, 1918, revoked by . . . . | 131 | | I | 1 |
| No 35, Sec. II, 1917, revoked by . . . . | 199 | | IV | |
| No 35, 1918. amended by. . . . . . . . | 43 | | III | |
| No 35, Par. 9, 1918, amended by . . . . | 77 | | VI | |
| No 35, Par. 5, 1918, amended by . . . . | 130 | | III | 1 |
| No 35, Par. 13, 1918, revoked by . . . . | 133 | | VI | |
| No 36, Par. 3, 1917, revoked by . . . . | 96 | | I | 4 |
| No 38, Par. 9, 1917, amended by . . . . | 19 | | II | |
| No 38, Pars. 11-12, 1917, amended by. . | 37 | | I | |
| No 38, Sec. II, 1917, rescinded by . . . | 105 | | III | |
| No 38, 1918, amended by . . . . . . . | 88 | | I | |
| No 38, Sec. I, 1918, supplemented by. . | 139 | | V | |
| No 39, 1917, revoked by . . . . . . . . | 122 | | II | |
| No 40, Sec. II, Par. 7, 1917, amended by. | 2 | | III | |
| No 40, Par. 2, 1917, amended by . . . . | 8 | | III | |
| No 40, Sec. I, Par. 8-A, 1918, revoked by. | 77 | | VIII | |
| No 40, Sec. I, Pars. 6-8, 1918, amended by . . . . . . . . . . . . . . . | 100 | | I | 14 |
| No 40, Pars. 2-11, 1918, revoked by. . . | 170 | | IV | |
| No 41, Sec. I, Par. 5, 1918, amended by. | 88 | | III | |
| No 41, Sec. I, Par. 8, 1918, amended by. | 100 | | I | 14 |
| No 41, Article 11, 1918, revoked by. . . | 150 | | III | 1 |
| No 42, Sec. II, Par. 2, 1917, amended by. | 63 | | IV | |
| No 43, 1917, revoked by. . . . . . . . | 44 | | | |
| No 43, Sec. II, 1918, if conflicting, revoked by . . . . . . . . . . . . . . | 180 | | III | 20 |
| No 43, 1918, if conflicting, revoked by. . | 111 | | XI | 15 |
| No 43, Sec. II, 1918, amended by. . . . | 58 | | II | |
| No 43, Sec. IV, 1918, amended by . . . | 195 | | I | |
| No 43, Sec. V, Pars. 1-2-3, 1918, revoked. | 186 | | II | |
| No 44, Sec. II, 1917, modified by. . . . | 154 | | III | |
| No 45, Sec. IV, 1918, amended by . . . | 155 | | III | |
| No 45, Sec. V, 1918, revoked by . . . . | 171 | | II | 2 |
| No 46, Sec. I, par. 3-(c), 1917, amended by . . . . . . . . . . . . . . . | 156 | | II | |
| No 46, Sec. I, par. sub. 3, 1917, amended by . . . . . . . . . . . . . . . | 58 | | I | 1 |
| No 46, Sec. II, par. 1, 1917, amended by. | 54 | | II | |
| No 46, Sec. III, par. 6, 1917, revoked by. | 36 | | I | |
| No 46, 1918, revoked by . . . . . . . . | 111 | | I | 1 |

| PURPORTS | G. O. | Bul. | Soc. | Par. |
|---|---|---|---|---|
| **General Orders** (Cont'd) — | | | | |
| No 48, Sec. I, 1917, amended by . . . . | 15 | | II | |
| No 49, 1918, supplemented by . . . . . | 102 | | | 6 |
| No 50, 1917, if conflicting, revoked by . | 5 | | I | 8 |
| No 50, 1918, Re R. R. and C., extended by. | 78 | | IV | 7 |
| No 50, Sec. II, 1918, amended by. . . . | 89 | | II | |
| No 51, Sec. II, 1918, revoked by. . . . | 175 | | | |
| No 51, Sec. III, 1918, revoked by. . . . | 181 | | I | 1 |
| No 52, Sec. II, 1918, revoked by . . . . | 80 | | I | |
| No 52, Sec. III, 1918, if conflicting, revoked by . . . . . . . . . . . . . . | 186 | | II | |
| No 53, Par. 2. 1917, amended by. . . . | 78 | | II | |
| No 53, Sec. II, 1918, if conflicting, revoked by . . . . . . . . . . . . | 91 | | II | |
| No 53, Sec. III, 1918, revoked by. . . . | 199 | | IX | |
| No 54, Sec. III, 1917, amended by . . . | 15 | | II | |
| No 54, Sec. II, 1918, partly revoked by . | 152 | | V | 2 |
| No 56, Sec. II, 1918, revoked by . . . . | 167 | | I | |
| No 58, 1917, supplemented by . . . . . | 102 | | | 6 |
| No 60, Par. 3, 1917, supplemented by. . | 159 | | IV | |
| No 62, 1917, supplemented by . . . . . | 24<br>45 | | II<br>I | |
| No 63, Par. 3, 1917, revoked by . . . . | 42 | | I | |
| No 63, Par. 4, 1917, amended by . . . . | 33 | | V | |
| No 64, Sec. III, 1917, revoked by. . . . | 9 | | I | 5 |
| No 65, Sec. III, 1918, amended by . . . | 102<br>120 | | <br>IV | 6<br> |
| No 66, Sec. IV, 1917, if conflicting, revoked by . . . . . . . . . . . . . . | 32 | | III | 1-D |
| No 66, Sec. I, 1918, amended by . . . . | 69 | | III | |
| No 67, 1917, revoked by . . . . . . . . | 100 | | I | 15 |
| No 67, 1918, amended by. . . . . . . . | 82 | | I | |
| No 68, Par. 2, 1918, amended by. . . . | 100 | | I | 14 |
| No 68, Par. 4, 1918, revoked by . . . . | 170 | | IV | |
| No 68, Sec. I, 1918, extended by . . . . | 165 | | II | |
| No 70, 1917, revoked by. . . . . . . . | 74 | | | |
| No 70, 1918, supplemented by . . . . . | 144 | | VI | 1 |
| No 71, Par. 5, 1917, revoked by . . . . | 43 | | II | 1 |
| No 71, Sec. I, 1917, if conflicting, revoked by . . . . . . . . . . . . . . | 180 | | III | 20 |
| No 71, Sec. II, Par. 1, 1917, supplemented by.. . . . . . . . . . . . . . | 41 | | I | |
| No 72, 1918, partly amended by . . . . | 155 | | I | 2 |
| No 73, 1917, revoked by . . . . . . . . | 44 | | | |
| No 73, Par. 9, 1918, amended by . . . . | 155 | | II | |
| No 73, Sec. III, 1918, revoked by. . . . | 147 | | III | |
| No 74, Sec. I, Par. 3, 1917, amended by. | 16 | | I | |
| No 74, Sec. I, Par. 1, 1917, enlarged by. | 167 | | IV | 1 |
| No 74, Sec. II, 1917, supplemented by . | 41 | | II | |
| No 74, Par. 2, 1918, amended by. . . . | 114 | | I | 3 |
| No 77, Sec. VII, 1918, revoked by . . . | 170 | | IV | |
| No 78, Sec. III, 1918, revoked by. . . . | 146 | | | |
| No 79, Par. 3, sub. 5, 1918, supplemented by . . . . . . . . . . . . . . . . . | 107 | | | |
| No 79, Sec. III, Par. 1, 1918, amended by. | 93 | | I | |

| PURPORTS | G. O. | Bul. | Soc. | Par. |
|---|---|---|---|---|
| **General Orders** (Cont'd) — | | | | |
| No 79, Sec. I, Par. 1, 1917, revoked by. | 196 | | I | |
| No 80, Sec. II, Par. 2, 1917 . . . . . . } | 50<br>157 | | III<br>I | |
| No 81, Sec. I, 1918, supplemented by. . | 142 | | III | |
| No 81, Sec. III, Par. 5, 1918, revoked by. | 157 | | II | |
| No 81, Sec. IV, Par. 1, 1918, amended by. | 130 | | IX | 1 |
| No. 82, 1917, partly revoked by . . . . | 91 | | I | 5 |
| No 84, Par. 2, 1918, partly revoked. . . | 135 | | III | 3 |
| No 89, Sec. I, 1918, amended by . . . . | 130 | | IV | 1 |
| No 95, 1918, supplemented by . . . . . | 121 | | III | 3 |
| No 99, Sec. II, 1918, amended by. . . . | 105 | | IV | 1 |
| No 100, Par. 14, 1918, revoked by . . . | 170 | | IV | |
| No 100, Sec. I, Par. 3, 1918, amended by. | 174 | | III | |
| No 101, 1918, revoked by. . . . . . . . | 146 | | | |
| No 102, Par. 3-c, 1918, revoked by . . . | 114 | | II | |
| No 102, Pars. 5-6-7, 1918, supplemented by. | 115 | | II | 3 |
| No 104, 1918, amended by . . . . . . . | 159 | | II | a |
| No 105, Sec. I, Par. 3, 1918, amended by. | 161 | | II | |
| No 106, 1918, supplemented by. . . . . | 110 | | III | 2 |
| No 110, Par. 4, 1918, amended by . . . | 147 | | III | |
| No 111, 1918, amended by . . . . . . . | 131 | | IV | 5 |
| No 111, 1918, extended by . . . . . . . | 165 | | II | |
| No 111, Sec. VII, Par. 1, 1918, amended by. | 179 | | III | |
| No 111, Sec. XI, 1918, revoked by. . . . | 180 | | III | 20 |
| No 116, 1918, amended by . . . . . . . | 121 | | VI | |
| No 116, Sec. IV, 1918, revoked by . . . | 175 | | IV | |
| No 118, Sec. I, 1918, amended by. . . . | 155 | | IV | |
| No 120, Sec. II, 1918, partly revoked by. | 185 | | IV | |
| No 121, Sec. VII, 1918, if conflicting, revoked by . . . . . . . . . . . . . . | 146 | | | |
| No 124, 1918, rescinded by. . . . . . . | 162 | | I | |
| No 127, Sec. I, Par. 1, 1918, amended by. | 189 | | VI | |
| No 130, Sec. II, Par. 1, 1918, amended by. | 159 | | III | 1 |
| No 130, Sec. VI, 1918, revoked by . . . | 170 | | IV | |
| No 131, Sec. II, 1918, supplemented by. | 135 | | 'III-IV) | |
| No 132, Par. 4. (sub. par. J-K), 1918, revoked by . . . . . . . . . . . . . . | 199 | | IV | |
| No 132, Sec. I, Par. 4, 1918, enlarged by. | 167 | | III | |
| No 135, Sec. II, 1918, amended by . . . | 163 | | III | |
| No 139, Sec. IV, 1918, extended by . . | 142 | | VI | |
| No 139, Sec. II, 1918, revoked by . . . | 157 | | III | |
| No 142, Sec. II, 1918, revoked by. . . . | 182 | | IX | |
| No 142, Sec. III, 1918, revoked by . . . | 182 | | IX | |
| No 144, Sec. I, 1918, revoked by . . . . | 162 | | I | |
| No 144, Sec. VII, 1918, amended by. . . | 150 | | V | 1 |
| No 153, 1918, partly revoked by . . . . | 168 | | III | |
| No 157, Sec. I, par. 2 (sub. B) 1918, amended by. . . . . . . . . . . . . . | 177 | | III | |
| No 158, Sec. III, 1918, if conflicting, revoked by . . . . . . . . . . . . . | 180 | | III | 20 |
| No 161, Sec. I, 1918, revoked by . . . . | 188 | | I | |
| No 168, Sec. II, 1918, revoked by. . . . | 188 | | I | |
| No 170, Sec. II, 1918, amended by . . . | 178 | | II | |
| No 175, Sec. II, 1918, partly revoked by. | 185 | | IV | |

| PURPORTS | G. O. | Bul. | Sec. | Par. |
|---|---|---|---|---|
| **General Surgery —** | | | | |
| Senior Consultant in. . . . . . . . . . . | 88 | | | |
| **Genito-Urinary, Venereal and Skin —** | | | | |
| Senior Consultant in. . . . . . . . . . . | 88 | | | |
| **Government Property —** | | | | |
| Excess to be collected and shipped . . . . | 10 | | II | |
| **Grade, Reduction of —** | | | | |
| Soldiers evacuated from hospitals not to be reduced . . . . . . . . . . . . . . . . | 98 | | III | |
| **Graves, marking of, etc.** . . . . . . . . . . . | 30 | | | |
| | 122 | | | |
| **Graves Registration Service** . . . . . . . . | 30 | | IV | 1-6 |
| | 50 | | I | |
| | 40 | | I | 6 |
| | 89 | | | |
| | 101 | | | |
| **Hay —** | | | | |
| Issue of, as bedding. . . . . . . . . . . . | 41 | | VII | |
| **Heating —** | | | | |
| Rights for, in billets. . . . . . . . . . . | 18 | | | 30 |
| **Hospitals —** | | | | |
| Officers and soldiers in, classified in "D". | | | | |
| Regulations relating to same. . . . . . . | 161 | | I | |
| Reports of patients and personnel, form of. | 170 | | II | |
| **Hospital, Mess —** | | | | |
| Admission and charge of civilian employee patients. . . . . . . . . . . . . . . . | 53 | | III | |
| **Hospital and Centrally Heated Buildings —** | | | | |
| Fuel allowance . . . . . . . . . . . . . . | 117 | | I | 16 |
| **Hospital Mobile.** . . . . . . . . . . . . . . . | 70 | | II | |
| **Identification Cards —** | | | | |
| Kept in possession by officers . . . . . . | 42 | | I | 2 |
| **Identification Numbers —** | | | | |
| Assignment to soldiers. . . . . . . . . . | 27 | | I | 1-5 |
| **Identification Tags.** . . . . . . . . . . . . . | 158 | | | |
| | 30 | | IV | 7 (n) |
| | 53 | | II | |
| To be carried on leave. . . . . . . . . . . | 6 | | | |
| | 91 | | | |
| Religion stamped on. . . . . . . . . . . | 122 | | III | 1 |
| **Immunizations —** | | | | |
| Complete service records of . . . . . . . | | 34 | | |
| **Income Tax —** | | | | |
| Returns. . . . . . . . . . . . . . . . . | | 14 | II | |
| | | 64 | | |
| **Increased Pay —** | | | | |
| When officer succeeds to higher command by seniority . . . . . . . . . . . . . . . | | 23 | II | |
| **Indemnity for damages —** | | | | |
| Caused by the A. E. F. . . . . . . . . . . | 78 | | IV | |
| **Information —** | | | | |
| Re-enclosed Ford Ambulances . . . . . . . | 2 | | | |
| **Inspection —** | | | | |
| Before going on leave. . . . . . . . . . . | 6 | | | 11 |
| Of Animals . . . . . . . . . . . . . . . . | 122 | | II | 8 |

| PURPORTS | G. O. | Bul. | Sec. | Par. |
|---|---|---|---|---|
| **Inspectors General Medical —** | | | | |
| Announcement of | 29 | | IV | |
| **Instruction —** | | | | |
| Re remount and veterinary services | 122 | | II | 11 |
| **Interment of Enemy's Dead** | 50 | | I | |
| **Issue of Animals** | 122 | | II | 3 |
| **Issue of Hay** | 41 | | VII | |
| **Issue of Supplies by A. E. F. to Navy Dep't —** | | | | |
| For forces serving in France | 41 | | III | |
| **Italy —** | | | | |
| Leaves of troops in | 6 | | | 22 |
| **Judge Advocate General, Acting —** | | | | |
| Appointee | 66 | | VI | |
| **Judge Advocate General's Office —** | | | | |
| Establishment of branch for A. E. F | 37 | | III | |
| **Jute Bags, Use in Shipment of Bread** | 20 | | I | 1-5 |
| **Kitchens, Field Plans for** | 11 | | IV | 8 |
| **Kitchens Rolling, Additional Allowance** | 92 | | | 2-3 |
| Fuel allowance | 117 | | I | 7 |
| **Knights of Columbus —** | | | | |
| Militarized members | 33 | | V | |
| Sales of subsistence authorized to members of | 159 | | II | |
| **Labor Local —** | | | | |
| Employment of | 32 | | III | |
| Request for | 131 | | V | |
| **Labor —** | | | | |
| Requisitioning of | 18 | | | 53-75 |
| **Laundries —** | | | | |
| To be provided and operated by Q. M. C. | 13 | | I | 1 |
| Re personnel of | 60 | | III | 1 |
| Re Fuel allowance | 117 | | I | 12-13 |
| **Laundries Work —** | | | | |
| Not to be done for | 13 | | I | 4 |
| **Leases —** | | | | |
| Through Town Major and Service de l'Intendence | 18 | | | 45 |
| **Leaves of Absences** | 6<br>28<br>29<br>38 | | | |
| Bureau of, for Officers | | 62 | | |
| To excepted Zones how obtained | 163 | | | |
| To designated areas, transportation by Q. M. C. | 139 | | | |
| When granted to excepted zones, how to forward request | 135 | | | |
| **Liaison —** | | | | |
| Officers, French, exercising right of requisitioning through | 18 | | | 72 |
| Service, organization of | 28 | | | 1-6 |
| System of, between French Gov't. and A. E. F | 8 | | II | |
| With French Military Service | 61 | | | 5 |

| PURPORTS | G. O. | Bul. | Sec. | Par. |
|---|---|---|---|---|
| **Liberty Loan —** | | 18 | II | |
| Third | 59 | | | |
| Fourth | 164 | | | |
| Transfer of | | 6 | | |
| Who may purchase same | 164 | | | |
| **Lighting plants** | 33 | | | 1 |
| **Local Purchases by Q. M. Eng. and Ordnance** | 32 | | III | |
| **Lodging** | 6 | | | 18 |
| Reports | 117 | | V | |
| **Long Distance Calls —** | | 48 | I | |
| **Lumber —** | | | | |
| Economy in use of | | 28 | | |
| **Machinery —** | | | | |
| For repair to be sent at Salvage Service | 10 | | III | |
| **Mail —** | | | | |
| Censorship regulations (Revoking G. O. 13, G. H. Q. 1917) | 146 | | | |
| **Mail Orderly Service —** | | | | |
| Establishment of | 61 | | | 5 a |
| **Mange** | | 8 | | |
| **Maps —** | | | | |
| Method of obtaining | 68 | | IV | |
| Instructions concernings maps | 142 | | IV | |
| **Matches** | 43 | | | 2 |
| **Material —** | | | | |
| Captured | 105 | | V | |
| Needed by A. E. F. | 60 | | | |
| Salvage of | 10 | | III | 6 |
| War, for troops in Italy, to be admitted free from duty | 17 | | III | |
| **Marine Corps —** | | | | |
| Service records to be sent to Paymaster U. S. Marine Corps, France | 165 | | | |
| **Maxillo-Facial Surgery —** | | | | |
| Senior Consultant in | 88 | | | |
| **Medical Inspectors, General —** | | | | |
| Announced | 29 | | IV | |
| **Medical Officers —** | | | | |
| Instructing men transporting patients on danger of gas heater poisoning in motor cars | 2 | | II | 6 |
| General order amended to change designation | 88 | | | |
| **Medical Property —** | | | | |
| Abandoned, and unserviceable, consignment to and repair by Medical Department | 10 | | III | 10 |
| **Medical Reports** | 100 | | | |
| **Medicine Charge not to be made** | 53 | | III | |
| **Men Enlisted —** | | | | |
| Absent without leave, forfeiture of pay | 16 | | II | |
| Articles withdrawn from sale to | 37 | | I | |
| Hunting by | 14 | | II | |

| PURPORTS | G. O. | Bul. | Sec. | Par. |
|---|---|---|---|---|
| **Men, Enlisted** (Cont'd) — | | | | |
| Leaves | 6 | | | |
| Money commutation for rations in travel | 32 | | II | |
| Rations for travelling | 34 | | IV | |
| Recommended for honors | 48 | | V | |
| Writing parents regularly | 16 | | V | |
| **Mess** — | | | | |
| Officers, rooms for | 60 | | | |
| Waste | 20 | | III | 1-3 |
| **Method of Supply in Rear of Advance Section** | 44 | | | 35 |
| **Mexican Service Badge** — | | | | |
| Other than authorized under O. O. 155 W. D. Wash. 1917, not to be worn | | 70 | | |
| **Military Police Corps** | 150 | | IV | |
| | 111 | | | |
| | 152 | | VI | |
| | 158 | | III | |
| **Military Postal Express Service** — | | | | |
| Establishment of | 72 | | | |
| **Mobile Hospital** | 70 | | II | 3 |
| **Mobile Surgical Unit** | 70 | | II | 4 |
| **Mobile Veterinary Hospital** | 113 | | | |
| **Money Commutations for Enlisted Men, re Rations** | 32 | | | |
| **Mounts** — | | | | |
| Shipment of private | | 3 | I | |
| **Motor Vehicles** — | | | | |
| By whom driven | 21 | | | 1 |
| Regulations governing | 43 | | IV | 1-4 |
| Speed of | 80 | | II | |
| The U. S. will take over all privately owned motor transportation | 130 | | V | |
| Use of, instead of animal drawn | 66 | | IV | |
| Worn or damaged | 58 | | IV | 2 |
| **Muster Rolls** — | | | | |
| In the A. E. F. | | 22 | I | |
| **Music** — | | | | |
| Purchase of, for bands | | 73 | | |
| Allowance increased | | 73 | | |
| Initial allowance for newly recruited bands | | 73 | | |
| **Mustard Gas** — | | | | |
| Tar Paper a protection against | | 75 | | |
| **Name** — | | | | |
| Change of, Alien making request for naturalization, authorized to | | 77 | | |
| **National Army** — | | | | |
| Abolished, merged into U. S. A | 144 | | I | |
| **National Guard** — | | | | |
| Abolished, merged into U. S. A. | 144 | | I | |
| **Naturalization** — | | | | |
| Bureau of | | 68 | | |
| Of aliens, to show army serial number | 171 | | I | |

| PUKPORTS | G. O. | Bul. | Sec. | Par. |
|---|---|---|---|---|
| **Naturalization** (Cont'd) — | | | | |
| Request to change name upon application for. | | 77 | | |
| Regulations for . . . . . . . . . . . . . . | 151 | | I | |
| Re Forms for . . . . . . . . . . . . . . . | 151 | | | |
| **Neurological Surgery** — | | | | |
| Senior Consultant in. . . . . . . . . . . | 88 | | | |
| **Neuro-Psychiatry** — | | | | |
| Senior Consultant in . . . . . . . . . . | 88 | | | |
| **Non-Commissioned Officers** — | | | | |
| Appointment of. . . . . . . . . . . . . | | 1<br>19 | | |
| **Nose, Ear, and Throat Surgery** — | | | | |
| Senior Consultant in. . . . . . . . . . . | 88 | | | |
| **Numerical Designation of Sanitary Squads** — | | | | |
| Changes in . . . . . . . . . . . . . . . | 153 | | | |
| **Number** — | | | | |
| Army Serial, of soldier, use of. . . . . . | | 49 | III | |
| **Numbering of soldiers** . . . . . . . . . . . | 27 | | | |
| **Nurse Corps** — | | | | |
| Army, rates for commutation of rations. | 30 | | VIII | |
| **Nurses** — | | | | |
| Army, leave of . . . . . . . . . . . . . | 6 | | | 20 |
| Accommodation and allowances . . . . . | 53 | | | |
| Accommodation space allowed to. . . . . | 58 | | | |
| To have laundry work done by Q. M. . . | 13 | | I | 1 |
| Travel allowance . . . . . . . . . . . . | 131 | | I | |
| **Octroi Dues** — | | | | |
| Toll on food staffs, Fodder, Fuel etc. . . | | 66 | | |
| Commutated to flat payment for A. E. F. | | 66 | | |
| Exceptions . . . . . . . . . . . . . . . | | 66 | | |
| Rules for computation. . . . . . . . . . | | 66 | | |
| **Offenses** — | | | | |
| Against French People, severe punishment for . . . . . . . . . . . . . . . . . . | 56 | | IV | |
| **Officers** — | | | | |
| Accountable, before discharge of the A.E.F., to obtain certificate of non-indebtedness. . . . . . . . . . . . . . . . . . | 127 | | | |
| Authorized to wear trench coat. . . . . . . | 7 | | | |
| Admitted to Allied Military hospitals, rations . . . . . . . . . . . . . . . . | 77 | | II | |
| Accountable for property, to prepare final returns, upon reaching destination. . . | 167 | | IV | |
| And soldiers, belonging to organizations serving in France and England, who are absent from the country in which their respective units are serving and whose transfers are otherwise provided for in G. O. 46. | 68 | | | 2 |
| And soldiers transferred to a depot division or base division under the provisions of Sec. 3, G. O. 23, who are absent from France. . . . . . . . . . . . . . . | 68 | | | 6 |
| And soldiers not to disclose certain information when taking lodging. . . . . . | 117 | | V | 4 |

| PURPORTS | G. O. | Bul. | Sec. | Par. |
|---|---|---|---|---|
| **Officers** (Cont'd) — | | | | |
| And Non-Commissioned officers selected for army and school. . . . . . . . . . . | | 39 | | |
| And soldiers in hospitals, classified in "D" regulations relating to same. . . . | 161 | | | |
| And soldiers, French attached to the A. E. F., rate of pay etc.. . . . . . . . . | 157 | | | |
| And soldiers, serving with the A. E. F., death report of . . . . . . . . . . . . . | 178 | | II | |
| And soldiers with A. E. F., authorized sales of subsistence to. . . . . . . . . | 159 | | II | |
| And soldiers, of M. P. C., insignia to be worn on coat collar . . . . . . . . . . . | 152 | | | |
| And soldiers, inquiries to be made before joining organizations . . . . . . . . . . | 133 | | | |
| Caps, style of, to be worn by . . . . . . | 149 | | | |
| Cautioning men to write parents. . . . . | 16 | | V | |
| Charged with censorship of mail and those using censor stamp, to exercise care. . . . . . . . . . . . . . . . . . . . | | 75 | | |
| Chief liaison . . . . . . . . . . . . . . . . . | 28 | | | |
| Chief Ordnance, appointment of. . . . . | 69 | | | |
| Commanding, duty of collecting and forwarding unserviceable property to salvage . . . . . . . . . . . . . . . . . . . | 10 | | III | 2 |
| Change of duties and status of, reporting. | | 49 | I | |
| Disposition of, when reported adversely under G. O. 25, and 45 . . . . . . . . | 87 | | III | |
| Duty in seeing orders given are obeyed. . | 4 | | | 1-3 |
| Efficiency of, investigation. . . . . . . . | 45 | | | |
| Excepted from "Octroi Dues" when forming part of garrison. . . . . . . . . . | | 66 | | |
| Faults noted in same. . . . . . . . . . . . | 4 | | | 3 |
| Failure to pay premiums on War Risk Ins. | | 22 | II | |
| Fuel for quarters . . . . . . . . . . . . . | 117 | | I | 6 |
| Gas. . . . . . . . . . . . . . . . . . . . . | 79<br>107 | | | |
| Historical, rules governing same. . . . . | 156 | | | |
| Issue of arms to. . . . . . . . . . . . . . | 41 | | II | 1 |
| Issuance of clothing to, when received at hospital from front . . . . . . . . . . | 77 | | III | |
| Increased pay where promoted to higher command by seniority. . . . . . . . . | 23 | | II | |
| Leave Bureau. . . . . . . . . . . . . . . | | 62 | | |
| Liaison, American at French Regional Hq'ts. . . . . . . . . . . . . . . . . . . | 32 | | | |
| Making allotments for Liberty Loans, to make notation on their pay accounts. . | 164 | | | |
| Ordered by corps commanders to Casual Camp, Blois, for re-classification and assignment . . . . . . . . . . . . . . . | 117 | | II | |
| Overcharging by certain French storekeepers. . . . . . . . . . . . . . . . . . | | 33 | | |
| Prohibited wearing enlisted men's breeches and coats . . . . . . . . . . . . . . . | 7 | | | |

| PURPORTS | G. O. | Bul. | Sec. | Par. |
|---|---|---|---|---|
| **Officers** (Cont'd) — | | | | |
| Promotion to grade of Colonel inclusive requirements . . . . . . . . . . . . . . | 162 | | | |
| Quarters, dimension of room allowed. . . | 156 | | | |
| Recommended for honors . . . . . . . | 48 | | V | |
| Recommendation of, for promotion. . . . | 124<br>162 | | | |
| Receipting for rations received from Allied forces . . . . . . . . . . . . . . . . . | 32 | | IV | |
| Railroad transportation, duties of. . . . . | 17 | | I | 2 |
| Regulating, assignment and duties. . . . | 20 | | IV | |
| Regulations re promotions. . . . . . . . | 144 | | | |
| Responsible for transportation of patients, to comply with. . . . . . . . . . . . | 2 | | | |
| Returning to U. S. report to A. G. of Army. | 63 | | | |
| Replacement depot, established . . . . . | 58 | | III | |
| Replacement depot, organization of. . . . | 105 | | III | |
| Sent to the U. S. for duty with new divisions to comply with . . . . . . . . . . | 168 | | | |
| To present a neat and soldierly appearance . . . . . . . . . . . . . . . . . . | 7 | | | |
| To be issued enlisted men's uniform and equipment. . . . . . . . . . . . . . . . | 7 | | | |
| To be issued fatigue clothing. . . . . . . | 7 | | | |
| Tardiness to work. . . . . . . . . . . . . | 4 | | | 1-3 |
| Temporary, . . . . . . . . . . . . . . . | 24 | | | |
| To deduct from pay accounts, rations furnished in the field. . . . . . . . . . . | 121 | | IV | |
| Transfer to tank corps. . . . . . . . . . | | 23 | | |
| Uniform, Overseas cap adopted. . . . . . | 7 | | | |
| Unfit. . . . . . . . . . . . . . . . . . | 24 | | | |
| Visiting front lines, instructions for . . . | 29 | | I | |
| Veterinary, reporting. . . . . . . . . . . | 122 | | II | 1-10 |
| Who examine or stamp mail, instructions for . . . . . . . . . . . . . . . . . . . | 146 | | | |
| **Offices** — | | | | |
| Renting of . . . . . . . . . . . . . . . | 18 | | | 47 |
| **Oil** — | | | | |
| Economy in use of. . . . . . . . . . . . | 19 | | I | 1 |
| **Ophthalmology** — | | | | |
| Senior consultant in. . . . . . . . . . . | 88 | | | |
| **Organization** — | | | | |
| Tables of. . . . . . . . . . . . . . . . | 67 | | | |
| **Organization of G. H. Q. A. E. F.** (**Table**). . . | 31 | | | |
| **Organization of Liaison Service**. . . . . . . | 28 | | | |
| **Orthopedic Surgery** — | | | | |
| Senior consultant in . . . . . . . . . . . | 88 | | | |
| **Outfit** — | | | | |
| Shoe repairing . . . . . . . . . . . . . | 58 | | V | 2 |
| **Overseas Caps** — | | | | |
| Adopted. . . . . . . . . . . . . . . . . | 7 | | I | 6 |
| For soldiers etc. . . . . . . . . . . . . | 7 | | I | 7 |
| For Officers. . . . . . . . . . . . . . . | 7 | | I | 8 |
| **Packages** — | | | | |
| Censorship regulations. . . . . . . . . . | 146 | | | |

| PURPORTS | G. O. | Bul. | Sec. | Par. |
|---|---|---|---|---|
| **Packages** (Cont'd) — | | | | |
| Rules re sending of same to members of the A. E. F. | 166 | | I | |
| **Paper** — | | | | |
| Economy in use of. | 48<br>83 | | IV<br>II | |
| **Parcels for Members of A. E. F., shipment of.** | | 24 | | |
| **Patients** — | | | | |
| In hospitals to have laundry work done Q. M. | 13 | | I | 1 |
| Transportation of, in gas heated ambulances. | 2 | | II | |
| **Pay** — | | | | |
| Enlisted man's, required to be allotted | 16 | | II | |
| **Paymaster** — | | | | |
| Authorized to issue travel orders. | 105 | | III | |
| Chief, U. S. Marines. | 16 | | II | |
| **Payments** — | | | | |
| Duplication of. | 78 | | IV | |
| **Pay Record Book** — | | | | |
| Adopting of an individual | 126 | | | |
| **Pay Rate** — | | | | |
| Determination of | 50 | | III | |
| **Payrolls** — | | | | |
| Preparation of | | 22<br>31 | | |
| **Payeurs** | 61 | | | 5 (b) |
| **Permits** — | | | | |
| Workers | 25 | | I | 1 |
| **Personnel Clerks** — | | 22 | I | |
| **Personnel** — | | | | |
| And material of dental allowances for combat divisions. | 99 | | I | |
| Bureau of. | | 94 | | |
| Replacement of. | | 46 | | |
| Replacement requisition, instructions re. | 148 | | | |
| **Petroleum** — | | | | |
| Economy in use of. | 19 | | I | 1 |
| **Photographs** — | | | | |
| Not to be taken. | 15 | | I | 4 |
| Of Medical Department | 78 | | III | |
| **Portable Disinfectors, Laundries and Bathing Units, Personnel of.** | 60 | | III | |
| **Postal Express Service** — | | | | |
| Military. | 72<br>155 | | | |
| **Postal Service** | 61 | | | 5 |
| **Prices** — | | | | |
| Prescribed, observance of | 6 | | I | 11 |
| **Prisoners** — | | | | |
| Attending drill | 4 | | | 2 |
| Investigation of mental responsibility. | 56 | | | |
| Maintenance of inquiry bureau. | 100 | | | |
| Of war. | 106<br>159 | 60<br>62 | | |

| PURPORTS | G. O. | Bul. | Sec. | Par. |
|---|---|---|---|---|
| **Promotions** — | | | | |
| In general | 124<br>162 | | | |
| Ratio of, to replacements | 56 | | VIII | |
| Recommendations | 24<br>124<br>162<br>135 | | | |
| Regulations re officers | 144 | | | |
| To grade of Colonel inclusive | 162 | | | |
| **Property** — | | | | |
| Accountability, officers to prepare final returns | 167 | | | |
| Accounting, surveying officer, during present emergency, suspended and procedure substituted therefore | 133 | | | |
| Accountable officer, before discharge of the A. E. F., to comply with | 127 | | | |
| Claims for loss or damage of, by officers and enlisted men | | 52 | | |
| Civilian, damage of | | 24 | II | |
| Disposition of, when division or organization leaves training area | 55 | | | |
| Engineer | | 3 | II | |
| French, damage to, by troops | 18 | | | 11 |
| For troops | 16 | | | |
| Of dead officers and men | 40 | | I | 5 |
| Personal, disposition of | 68 | | | 4 |
| Purchase of, in Europe | 16 | | | 6-9 |
| Returns of | 16<br>167 | | | |
| **Prophylactic and Censor Station** | 6 | | | |
| **Protection Against Mustard Gas** | | 75 | | |
| **Provost Marshal General** — Duties of | 180 | | | |
| **Purchases** — | | | | |
| Of food supplies | | 33 | | |
| Of music for bands | | 73 | | |
| Of timber | 130 | | | |
| **Purchasing Officer** — | | | | |
| Chief, rendering monthly abstracts of purchases to C. O. O. S. O. S. revoked | 42 | | II | |
| **Quartering and Billeting** — | | | | |
| Of troops | 18 | | | |
| In general | 18 | | | |
| **Quatermaster** — | | | | |
| Information furnished re Octroi Dues | | 66 | | |
| Representatives from, in office of the Chief Salvage Officer | 10 | | III | 4 |
| Remount Service attached | 122 | | II | 3 |
| Responsive for Salvage Service | 10 | | III | 1 |
| Sales to enlisted men | 37 | | | |
| Will provide and operate laundries | 13 | | | 1 |
| **Quarters** — | | | | |
| Officers, dimensions of space allowed | 156 | | | |
| Commutation of, to Field Clerks | | 78 | | |

| PURPORTS | G. O. | Bul. | Sec. | Par. |
|---|---|---|---|---|
| **Railheads and Refilling Points** . . . . . . . | 44 | | | 20-21 |
| **Railroad Transportation Officers** — | | | | |
| Duties. . . . . . . . . . . . . . . . . . | 17 | | I | 2 |
| Location of . . . . . . . . . . . . . . . | 17 | | I | 2 |
| **Raincoat** — | | | | |
| Authorization for Officers to wear, and for insignia on . . . . . . . . . . . . . . | 7 | | I | 2 |
| **Rates** — | | | | |
| Tariff, revocation of. . . . . . . . . . . | | 20 | II | |
| **Rations** — | | | | |
| Additions to garrisons. . . . . . . . . . | 84 | | | |
| Civilian employees, allowance of, when admitted to hospitals . . . . . . . . . | 53 | | III | |
| Commutation of, to enlisted men, drivers, couriers. . . . . . . . . . . . . . . . | 167 | | | |
| Drawing from Allied Forces. . . . . . . | 32 | | IV | |
| Distribution, methods of. . . . . . . . . | 44 | | | 25 |
| Forage, prescribed for animals in A. E. F. | 174 | | | |
| For animals. . . . . . . . . . . . . . . | 79 | | III | |
| Increased certain components of . . . . . | 50<br>11 | | | |
| Increase to troops engaged in manual labor. | 70 | | I | |
| Money commutation for, to enlisted men travelling . . . . . . . . . . . . . . . | 32 | | II | |
| Payments to be made on certificate of commissioned officers . . . . . . . . . | 63 | | IV | |
| Officers and Soldiers . . . . . . . . . . | 54 | | | |
| Re increase for soldiers sick in hospitals . | 30 | | VIII | |
| Re deductions from pay account where furnished officers in the field. . . . . . | 121 | | IV | |
| Substitutive equivalent articles. . . . . . | 176 | | | |
| To be paid under conditions and at rate specified herein . . . . . . . . . . . . | 132 | | | |
| **Reciprocal Supplies Bureau** — | | | | |
| Establisment of . . . . . . . . . . . . . | 152 | | | |
| **Record Book, Pay**. . . . . . . . . . . . . . | 126 | | | |
| **Record of Animals** — | | | | |
| By veterinary service . . . . . . . . . . | 122 | | II | 7 |
| **Red Cross** — | | | | |
| American, assignment of officer and clerks to staff of Graves Registration Service . | 30 | | IV | 7 (m) |
| Militarized members . . . . . . . . . . . | 33 | | V | |
| Sales of subsistence authorized for members of . . . . . . . . . . . . . . . . | 159 | | | |
| Service, with the G. R. S. . . . . . . . . | 101 | | | |
| With the Graves Registration Service. . . | 101 | | | |
| **Regular Army** — | | | | |
| Merged into U. S. A. . . . . . . . . . . . | 144 | | | |
| **Regulating Officer** — | | | | |
| Assignment . . . . . . . . . . . . . . . | 20 | | IV | |
| Duties of . . . . . . . . . . . . . . . . . | 44 | | | 17 |
| Stamping date and hour of passage of men on leave. . . . . . . . . . . . . . . . | 6 | | | 16 |
| **Regulating Station** — | | | | |
| Definition of. . . . . . . . . . . . . . . | 44 | | | 15 |

| PURPORTS | G. O. | Bul. | Sec. | Par. |
|---|---|---|---|---|
| **Regulating Station** (Cont'd) — | | | | |
| Equipment to be left at | 15 | | III | |
| French | 61 | | | |
| Relieving enlisted men on leave of rifles, pistols and bayonets | 25 | | V | 7 |
| **Renting, Requisition and Claims Service** — | | | | |
| Renting and billeting of troops | 18<br>50<br>60<br>78 | | <br>IV<br>II<br>IV | |
| **Repair of Shoes** | 58 | | V | |
| **Repair of Materials** — | | | | |
| Of Salvage Service<br>Of Gas Service<br>Of Medical Department<br>Of Ordnance Department | 73 | | II | |
| **Replacement Depot** — | | | | |
| Officers, organization of | 58 | | III | |
| **Replacement of Personnel** | 111 | | | |
| **Replacement Requisitions** | 111<br>148 | | | |
| **Reports** — | | | | |
| And information, if possible to be sent by courier, officers of A. E. F., directed to use telegraph lines for matters of vital importance only | 131 | | | |
| Change of duties and status of officers | | 49 | I | |
| Efficiency | 39 | | I | 1-4 |
| Of burials | 50 | | I | |
| Of casualities | 169 | | | |
| Of deaths | 141 | | | |
| Of deaths of officers and soldiers | 170 | | | |
| Of lodgings under French Law | 117 | | V | |
| Of Veterinary Officers | 122 | | II | |
| Upon completion of courses | 35 | | | 14 |
| **Requisitions** — | | | | |
| Not to be made where surplus exists | 10 | | II | |
| Military | 18 | | | |
| Replacement | 46 | | III | |
| Receipts should be given | 60 | | | |
| Unfilled, notification concerning | 44 | | | 30 |
| **Respirators** — | | | | |
| Issuance of | 78 | | II | |
| Salvage of, by gas service | 28 | | II | 2 |
| **Retirement of enlisted men of A. E. F.** | | 31 | II | |
| **Roentgenology** — | | | | |
| Director of | 36 | | III | |
| Senior consultant in | 88 | | | |
| **Sales** — | | | | |
| Articles withdrawn from sale to enlisted men | 37 | | | |
| Of subsistence, for personal use of Officers and enlisted men, Red Cross, Y. M. C. A., etc. | 159 | | | |
| Of articles of subsistence required for sick in hospitals discontinued | | 67 | | |

| PURPORTS | G. O. | Bul. | Sec. | Par. |
|---|---|---|---|---|
| **Sales** (Cont'd) — | | | | |
| Of subsistence, authorized for members of Salvation Army, Red Cross, Y. M. C. A. and als | 159 | | | |
| Of subsistence | 104 | | | |
| **Sales Stores** — | | | | |
| To be established | 37 | | I | 3 |
| Rolling to be arranged for | 37 | | I | 4 |
| **Saluting** | 105 | | | |
| **Salvage Depots** — | | | | |
| Lines of communication, organization of | 10 | | III | 12 |
| Not to accept serviceable clothing or supplies | 34 | | V | |
| **Salvage Service** — | | | | |
| Organization of | 10<br>55 | | <br>II | |
| Materials to be repaired by | 73 | | II | |
| **Salvation Army** — | | | | |
| Militarized members | 33 | | V | |
| **Sam Browne Belt** — | | | | |
| Wearing of, prohibited in the U. S. | | 42 | | |
| **Sanitary Precautions** — | | | | |
| Instructions to cooks and kitchen help | | 57 | | |
| **Sanitary Squads** — | | | | |
| Changes in numerical designations | 97<br>115<br>125<br>153 | | | |
| Numbering of | 85 | | III | |
| **Schools** — | | | | |
| Army Candidates | 32 | | | |
| Army Corps | 35 | | | |
| Aeronautical | 35 | | | |
| Base training | 47 | | | |
| Eligibility of candidates | 32 | | | 2 |
| Engineer | 35 | | | |
| Establishment of | 32 | | | |
| For chauffeurs | 47 | | | |
| For company and regimental clerks, | 47 | | | |
| For company and battery mechanics | 47 | | | |
| For drivers | 47 | | | |
| For saddlers, horseshoers | 47 | | | |
| For signalers | 47 | | | |
| For mess sergeants, cooks and bakers | 47 | | | |
| Gas defense | 79 | | II | |
| Gas | 35 | | | |
| Infantry | 35 | | | |
| Second corps | 14 | | | |
| **Selective Service Regulations** | | 7 | | |
| **Senior Consultant in General Medicine** | 88 | | | |
| **Service** — | | | | |
| Engagement of (local) | 32 | | III | 2 |
| Establishment of | 31 | | | |
| Establishment of Q. M. C. sales stores by | 37 | | I | 3 |
| Exceptionally meritorious | | 25 | | |

| PURPORTS | G. O. | Bul. | Sec. | Par. |
|---|---|---|---|---|
| **Service** (Cont'd) — | | | | |
| Garden | 34 | | | |
| Hat | 7<br>117 | | I<br>III | |
| Inspection | 52 | | III | |
| Mail orderly, establishment of | 61 | | | 5 (a) |
| Of supply | 31 | | | 3 |
| Of veterinarian | | 21 | II | |
| Postal | 61 | | | 5 |
| Renting Requisition and Claims | 50 | | IV | |
| Salvage | 55 | | | |
| **Service Chevrons** — | | | | |
| Term of service | 73 | | III | |
| War | 26 | | I | |
| Wearing of | 33 | | IV | |
| **Service Records** | 49<br>165 | | II | |
| **Shoeing of animals** | 65 | | IV | |
| **Shoes** — | | | | |
| Of sick animals | 12 | | III | |
| Soldiers, repair of | 58 | | V | |
| **Sick and Wounded** — | | | | |
| Transfer of | 37 | | | |
| Under treatment in hospitals A. E. F., excepted from "Octroi Dues" | | 66 | | |
| **Signatures** — | | | | |
| Disbursing officers, specimens to be furnished | 37 | | IV | |
| **Soldiers** — | | | | |
| Army serial number of | 49 | | III | |
| And French Officers, attached to A. E. F., rate of pay, etc. | 157 | | | |
| Incapacitated, instruction before being returned to the States | | 26 | | |
| Instruction re trial of, General Court-martial | | 8 | | |
| Not permitted on pass unless | 7 | | I | 1 |
| On duty at recreation or leave camp | 38 | | I | 1 |
| Or any member of A. E. F. when captured instructed to communicate with A. R. C. at Berne, Switzerland | 127 | | | |
| Or officers in hospitals, and classified in "D", regulations relating to same | 161 | | | |
| On leave, to designated areas, transportation to, by Q. M. | 139 | | | |
| Service records of, dropped from roll of organization | 49<br>165 | | II | |
| **Special Reserve Ration** | 176 | | | |
| **Stamps, Censor** | 34<br>146 | | VII | |
| **Statistical Division** — | | | | |
| Section, duties of | 29<br>100<br>174 | | V | |
| **Summary Court** | 40 | | I | 7 |

| PURPORTS | G.O. | Bul. | Sec. | Par. |
|---|---|---|---|---|
| **Subsistence** — | | | | |
| Authorized sale of | 159 | | | |
| Articles of, required for sick in hospitals discontinued | | 67 | | |
| To be paid under conditions and at rate specified herein | 132 | | | |
| **Supplies** — | | | | |
| Bureau of Reciprocal, established | 152 | | | |
| Board of Allied Supply, appointed | 100 | | III | |
| Exchanges for | 32 | | III | |
| Footwear | 58 | | V | |
| French estimates of cost of, limit of time for presenting | 8 | | I | 1-2 |
| In general | 44 | | | |
| Issue of, by A. E. F., to Navy Depot | 41 | | III | |
| Needed by the A. E. F. | 60 | | | |
| Quartermaster | 104 | | | |
| Requisitioning of | 18 | | | 53-75 |
| Repair of, to be sent to salvage L. O. C. | 10 | | III | 13 |
| Supplies local, limit to be bought | 32 | | III | |
| **Supply Depots** — | | | | |
| Advance, return of excess equipment | 10 | | II | |
| Serviceable articles to be sent here | 34 | | V | |
| **Supply of Replacements** | 46 | | IV | |
| **Surgical Research** — Senior consultant in | 88 | | | |
| **Suspension of sentences** — | 118 | | I | |
| **Surveying Officer** — | | | | |
| During the present emergency, suspended, and procedure substituted therefore | 133 | | | |
| **Tags** — | | | | |
| Identification, rules re wearing of | 6<br>158 | | | 10 |
| Religion stamped on | 122 | | III | 1 |
| When and how worn | 30 | | IV | 7 (n) |
| Amended | 91 | | | |
| Amended | 53 | | II | |
| **Tallow** | | 6 | | |
| **Tax Return** — | | | | |
| Income | | 64 | | |
| **Telegraph Censorship Regulations** | 146 | | | |
| **Telegraphic Communications** | 86<br>155 | | | |
| **Telephone Censorship Regulations** | 146 | | | |
| **Telephone Service** | | 4 | I | |
| Obtained from French Administration | | 67 | | |
| Long distance calls | | 48 | I | |
| Restrictions governing use of | | 35 | I | |
| **Throat, Ear, and Nose Surgery** — | | | | |
| Senior consultant in | 88 | | | |
| **Timber** — | | | | |
| Economy in use of | | 28 | | |
| Rules and Regulations for purchase of | 130<br>159 | | | |
| **Tires** — | | | | |
| Unserviceable | 58 | | IV | 4 |

| PURPORTS | G. O. | Bul. | Sec. | Par. |
|---|---|---|---|---|
| **Tracing of Freight Cars** | 17 | | I | 6 |
| **Transportation Department** — | | | | |
| Duties of | 105 | | VI | |
| **Transportation of Patients** — | | | | |
| Care to be taken | 2 | | II | |
| **Transportation** — | | | | |
| For officers and men | 23 | | II | |
| In Ford Ambulances, precautions | 2 | | | |
| Horse drawn vehicles | 66 | | IV | 4 |
| Motor, care to be exercised | 66 | | IV | 1 |
| Motor, for other than military purposes strictly forbidden | 19 | | I | 1 (b) |
| On regular trains | 6 | | | 15 |
| On special trains | 6 | | | 14 |
| Travel on leave | 6 | | | 15 |
| **Travel Allowance** — | | | | |
| To be paid under conditions and at rate specified herein | 132 | | | |
| **Travel Ration** | 176 | | | |
| **Treatment and Control of Prisoners of War** | 159 | | | |
| **Trench Foot** | 11 | | | |
| **Underclothing** — | | | | |
| Exchange of clean, for soiled, by Q. M. | 13 | | | 1-2 |
| **Unit** — | | | | |
| Mobile surgery | 70 | | II | 4 |
| **Unserviceable solid tires, tubes, pneumatic casings** | 58 | | IV | 4 |
| **Use of French Telephone and Telegraph System** | | 28 | | |
| **Vacancies** — | | | | |
| Definition of term, re promotions of officers up to grade of Brig. Gen. | 162 | | | |
| **Venereal Disease** — | | | | |
| Facts about | | 54 | | |
| **Veterinary Officers** — | | | | |
| Casual, report to Chief Surgeon, A. E. F. | 122 | | II | 9 |
| Personal reports of | 122 | | II | 10 |
| Service of | | 21 | II | |
| **Veterinary Service** | 122<br>139 | | II | |
| **Visiting Correspondents** | 96 | | | |
| **Wage Scale** — | | | | |
| Prevention of disturbance in | 33 | | III | |
| **Wagon Transportation** | 66 | | IV | |
| **War Risk Insurance** — | | | | |
| Change of address of Beneficiary to be forwarded thru certain channels | | 77<br>74 | | |
| Enlisted men who have made allotment on Form 1-B to notify Bureau of change in address of allotee | | 74 | | |
| Failure of officers to pays premiums | | 22 | II | |
| Premiums not affected by sentence of Courts-Martial imposing forfeiture of pay | | 47 | II | |
| Procedure to obtain refund of over deduction | | 78 | | |

| PURPORTS | G. O. | Bul. | Sec. | Par. |
|---|---|---|---|---|
| **War Risk Insurance** (Cont'd) — | | | | |
| Promotion of. . . . . . . . . . . . . . . | 16 | | II | |
| Re combat-division. . . . . . . . . . . . | 25 | | II | 173 |
| **War Savings Stamps.** . . . . . . . . . . . | 59 | | I | 20 |
| **War Service Chevrons.** . . . . . . . . . . | 26 | | | |
| Specifications. . . . . . . . . . . . . . . | | 18 | I | |
| Term of Service. . . . . . . . . . . . . . | 73<br>110 | | III | |
| Wearing of. . . . . . . . . . . . . . . . | 33 | | IV | |
| **Waste** — | | | | |
| Mess. . . . . . . . . . . . . . . . . . . | 20 | | III | 1-3 |
| Elimination of . . . . . . . . . . . . . | 70 | | | 4 |
| **Water** — | | | | |
| Provisions of, by Town Major . . . . . . | 18 | | | 7 |
| Water Supply. . . . . . . . . . . . . . . | 34<br>131 | 55 | VI | |
| **Worker's Permits** . . . . . . . . . . . . . | 25 | | I | 1 |
| **Wound Chevrons**. . . . . . . . . . . . . . | 26<br>33 | | | |
| **Y. M. C. A.** — | | | | |
| Franking privileges not extended . . . . | 15 | | II | |
| Militarized members. . . . . . . . . . . . | 33 | | V | |
| Personal package service. . . . . . . . . | | 17 | | |
| Sales of subsistence to . . . . . . . . . . | 159 | | | |
| **Zone of Advance** — | | | | |
| Issue to officers entering, the equipment of men . . . . . . . . . . . . . . . . . | 7 | | I | 5 |
| Salvage service . . . . . . . . . . . . . | 10 | | | |
| **Zone of the Armies** — | | | | |
| French rail transportation denied there for absence other than duty or leave. . | 6 | | | 1-4 |
| Special permission required for leave. . . | 6 | | | |
| **Zone of Interior** — | | | | |
| Re rail transportation . . . . . . . . . | 6 | | | |
| **Zone Major** — | | | | |
| Destination of. . . . . . . . . . . . . . . | 48 | | III | |

*Le Gérant* : O. PORÉE. Paris. — Imp. LAHURE, 9, rue de Fleurus.

